# Profound impact of sample processing delay on gene expression of multiple myeloma plasma cells

**DOI:** 10.1186/s12920-015-0161-6

**Published:** 2015-12-30

**Authors:** Tobias Meißner, Anja Seckinger, Kari Hemminki, Uta Bertsch, Asta Foersti, Mathias Haenel, Jan Duering, Hans Salwender, Hartmut Goldschmidt, Gareth J. Morgan, Dirk Hose, Niels Weinhold

**Affiliations:** Department of Internal Medicine V, University of Heidelberg, Heidelberg, Germany; Department of Molecular and Experimental Medicine, Avera Cancer Institute, 11099 North Torrey Pines Road, La Jolla, CA, 92037 USA; Division of Molecular Genetic Epidemiology, German Cancer Research Center (DKFZ), Heidelberg, Germany; Center for Primary Health Care Ressearch, Lund University, Malmo, Sweden; Department of Internal Medicine III, Klinikum Chemnitz, Chemnitz, Germany; Department of Hematology, University Hospital Essen, Essen, Germany; Department of Hematology and Oncology, Asklepios Hospital Hamburg Altona, Hamburg, Germany; National Center for Tumor Diseases, Heidelberg, Germany; Myeloma Institute, Little Rock, AR, USA

**Keywords:** Multiple myeloma, Gene expression profiling, Sample processing delay

## Abstract

**Background:**

Gene expression profiling (GEP) has significantly contributed to the elucidation of the molecular heterogeneity of multiple myeloma plasma cells (MMPC) and only recently it has been recommended for risk stratification. Prior to GEP MMPC need to be enriched resulting in an inability to immediately freeze bone marrow aspirates or use RNA stabilization reagents. As a result in multi-center MM trials sample processing delay due to shipping may be an important confounder of molecular analyses and risk stratification based on GEP data.

**Results:**

We compared GEP data of 145 in-house and 246 shipped samples and detected 3301 down-regulated and 3501 up-regulated genes in shipped samples. For 3994 genes we confirmed differential expression in an independent set of 85 in-house and 97 shipped samples. Differentially expressed genes were enriched in processes like ribosome biogenesis, cell cycle, and apoptosis. Among GEP based risk predictors the IFM-15 seemed to underestimate high risk in shipped samples, whereas the GEP70 and the EMC-92 gene signatures were more robust. In order to provide a tool to assess the “shipping effect” in public repositories, we generated a 17-gene predictor for shipped samples with a 10-fold cross validation error rate of 0.06 for the training set and an error rate of 0.15 for the validation set.

**Conclusion:**

Sample processing delay significantly influences GEP of MMPC, implying it should be avoided if samples were used for risk stratification.

**Electronic supplementary material:**

The online version of this article (doi:10.1186/s12920-015-0161-6) contains supplementary material, which is available to authorized users.

## Background

Multiple myeloma (MM) is characterized by the accumulation of monoclonal malignant plasma cells (PC) in the bone marrow (BM) resulting in bone destruction, renal impairment, immunosuppression and hypercalcemia [1]. The implementation of high dose-chemotherapy and the introduction of immunomodulatory agents and proteasome-inhibitors improved the outcome of MM patients [2, 3]. Nevertheless, MM remains a disease with an unpredictable clinical course mainly attributable to its composition of a variety of molecular subtypes with a distinct pathogenesis [4]. Gene expression profiling (GEP) has significantly contributed to the elucidation of the molecular heterogeneity of MM [4–6] and only recently it has been recommended for risk stratification [7]. The University of Arkansas for Medical Sciences (UAMS) total therapies 4 (low risk) and 5 (high risk) and the mSMART algorithm used by the Mayo Clinic [8] are examples of risk-adapted strategies based on molecular data. Importantly, prior to expression analyses MMPCs need to be enriched preventing the immediate freezing of bone marrow aspirates or the use of RNA stabilization reagents after removal from the patient. As a result in multi-center MM trials utilizing central sample processing delay due to shipment (“shipping delay”) may have an important impact on gene expression. In order to determine the impact of “shipping delay” on MMPC gene expression we analysed a set of 573 newly diagnosed German MM patients including 230 in-house and 343 shipped samples.

## Methods

### Expression data

The impact of sample shipment on gene expression was investigated in publicly available GEP data of newly diagnosed MM patients treated in the German-Speaking Myeloma Multicenter Group (GMMG) HD4 and MM5 trials. The trials were done in accordance with the Declaration of Helsinki (Version 1996) and approved by the ethics committee Heidelberg and the local ethics committees of all other participating centers. The names of the participating centers and the ethics committees that granted approval to this study can be found in the Additional file 1 “participating_centers.xls”. Written informed consent were obtained from patients for treatment, sample procurement and publication of research findings. The GEP datasets are deposited in ArrayExpress (accession number E-MTAB-2299) and Gene Expression Omnibus GSE19784. All samples had been processed in a central laboratory in Heidelberg and include 85 HD4 and 145 MM5 in-house and 97 HD4 and 246 MM5 shipped samples. External samples where usually shipped and processed within 24 hours whereas in-house samples were processed the same day. Further prediction of sample status was done on publicly available data deposited at Gene Expression Omnibus under the accession numbers GSE21349 (UK) and GSE24080 (UAMS) and the Multiple Myeloma Research Consortium (MMRC) Reference Collection downloaded from the Multiple Myeloma Genomics Portal (http://www.broadinstitute.org/mmgp/home). From the MMRC dataset we selected the samples that were marked as ’untreated’ (*n*=122).

### Expression and statistical analysis

Main analyses were undertaken using R (v3.0) software. As chip definition file (CDF) we used the Affymetrix U133 Version 2.0 plus array custom (CDF) (v16) mapping to Entrez genes (http://brainarray.mhri.med.umich.edu/Brainarray/Database/CustomCDF/). Expression data were normalized using GC-RMA. Unsupervised complete linkage hierarchical clustering was performed using centered Pearson correlation distance. Differential gene expression was assessed using empirical Bayes statistics in linear models for microarray data [9]. Predictor for shipment status was generated on the MM5 cohort using prediction analysis for microarrays (PAM) [10]. The predictor was saved using a documentation-by-value strategy [11] and subsequently applied to samples from the HD4, UK, UAMS and MMRC cohorts. Pathway enrichment analysis was done using WebGestalt [12]. The gene expression based proliferation index (GPI) [13], the French Intergroupe Francophone du Myelome (IFM) 15 score (IFM-15) [14], the GEP70 [15], the EMC-92 [16], as well as molecular classifications [5, 6] were obtained as previously described. Fisher’s exact test was used to compare the subgroup distribution between cohorts. Overall survival (OS) of patients treated in the GMMG HD4 trial was calculated from randomization until death from any cause. For the HD4 the trial design and patient characteristics have recently been described [17]. Estimation of OS distribution was performed by the method of Kaplan and Meier. The log-rank test was used for comparisons of OS curves. The Cox proportional hazards model was used to access the impact of prognostic factors. If applicable, results were corrected for multiple testing using the Benjamini-Hochberg method. In all statistical tests, an effect was considered statistically significant if the *P*-value of its corresponding statistical test was not greater than 5 %.

### Cytogenetic analyses

Fluorescence in situ hybridization and ploidy classification were performed as previously described [18].

## Results

### Impact of shipping on gene expression

**Fig. 1:**
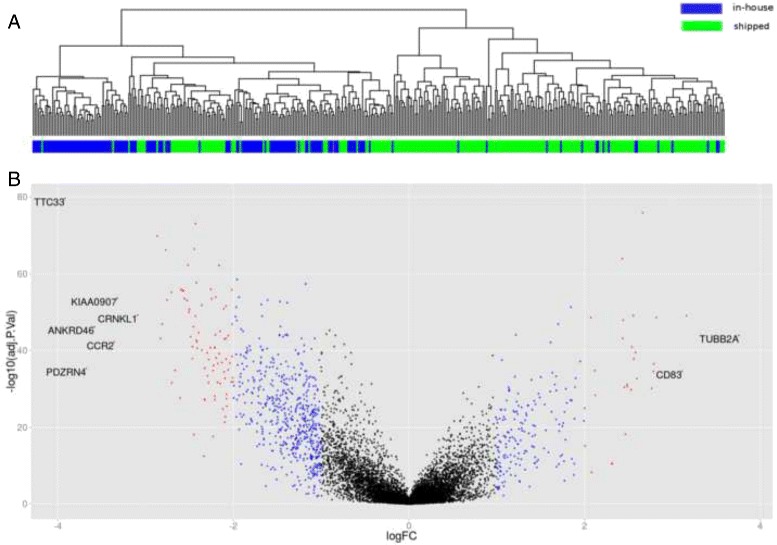
Impact of “shipping delay” on global gene expression. **a** Unsupervised hierarchical clustering showing separation into two distinct clusters. In-house samples colored in blue, shipped samples colored in green. **b** Volcano plot showing differential gene expression between 139 in-house and 252 shipped samples of the GMMG MM5 trial. The left-hand spread shows down-regulated genes in shipped samples. The right-hand spread shows the up-regulated genes. The color of each point reflects the log2 fold change (*logFC*) for the respective gene with absolute logFC <1 depicted in black, ≥ 1 & <2 in blue, and ≥ 2 in red. We added the gene symbol, if the absolute logFC was >3

We analyzed the impact of time delay between BM aspiration and cell sorting on gene expression in 391 samples of the GMMG MM5 multi-center trial including 145 in-house and 246 shipped samples. Applying the Goeman’s global test [19] on the MM5 set showed that “shipping delay” significantly impacted global gene expression (P <0.001). Unsupervised hierarchical clustering showed a separation into two main clusters. In-house and shipped samples were not evenly distributed across the two clusters with in-house and shipped samples showing an enrichment in cluster 1 and 2, respectively (P <0.001, Fig. 1a). A set of 6802 genes (40 %) were significantly differentially expressed between the two conditions. In shipped samples a total of 3301 genes were down-regulated and 3501 genes were up-regulated (Fig. 1b, Additional file 2: Table S1). Of these genes 2040 had a > 1.5-fold and 826 a > 2-fold difference in expression level. The highest fold change with a value of 13.5 was observed for *TUBB2A* that was up-regulated in shipped samples. The 10 most up- and down-regulated genes are presented in Table 1. Genes that were down-regulated in shipped samples showed an enrichment for 25 KEGG pathways (Additional file 3: Table S2). Up-regulated genes were enriched for 42 pathways (Additional file 3: Table S2). These included neurotrophin and the linked *MAPK* signaling pathways, in addition to many other signaling pathways. Among the 25 down-regulated pathways ribosome biogenesis and ubiquitin mediated proteolysis ranked on top; other pathways included RNA metabolism, many DNA repair pathways and the cell cycle. To investigate the impact of “shipping delay” on proliferation we applied the proliferation surrogate marker GPI to the MM5 set. This analysis showed significantly lower frequencies of medium (35 % vs. 60 %) or high (4.9 % vs. 13.8 %) proliferation rates in shipped samples (P <0.001).

**Table 1:** Ten most up- and down-regulated genes in shipped samples of the MM5 set

Up-regulated in shipped samples				Down-regulated in shipped samples			
Symbol	Entrez	Log2FC*	adjP**	Symbol	Entrez	Log2FC*	adjP**
*TUBB2A*	7280	3.76	1.18x10^−44^	*TTC33*	23548	3.92	2.63x10^−80^
LOC100506935**	1.01E+08	3.16	8.41x10^−50^	*PDZRN4*	29951	3.67	5.60x10^−36^
*CD83*	9308	3.11	2.50x10^−35^	*ANKRD46*	157567	3.58	7.05x10^−47^
*MAFF*	23764	2.82	2.32x10^−49^	*CCR2*	729230	3.36	6.52x10^−43^
*FAM209A*	200232	2.79	3.13x10^−37^	*KIAA0907*	22889	3.32	2.46x10^−54^
*NFKBIZ*	64332	2.78	3.03x10^−35^	*CRNKL1*	51340	3.09	6.01x10^−50^
*MAP3K8*	1326	2.76	7.95x10^−31^	*BBS10*	79738	2.86	1.68x10^−70^
*CSRNP1*	64651	2.66	1.22x10^−76^	*ZNF260*	339324	2.83	8.48x10^−44^
*NR4A2*	4929	2.59	2.24x10^−33^	*GOLPH3L*	55204	2.81	1.40x10^−47^
*MIR22HG*	84981	2.58	3.11x10^−40^	*TMEM68*	137695	2.77	5.87x10^−67^

^*^Log2FC: log2 of fold change, **Benjamini-Hochberg adjusted *P*-Value

^**^This record has been withdrawn by NCBI because the model on which it was based was not predicted in a later annotation

We examined the expression levels of the 6802 changed genes in an independent set of 182 patients (85 in-house, 97 shipped samples) treated in the GMMG HD4 trial. For 3994 (59 %) of these genes we could confirm changed expression levels in the same direction (data not shown). In this set, shipped samples also showed less medium (46.4 % vs. 58.1 %) or high (4.1 % vs. 9.3 %) proliferation rates (*P*=0.04).

A stratified analysis of the MM5 set by ploidy (status was available for 383/391 cases) showed that 4829 and 4624 genes were significantly differentially expressed between in-house and shipped hyperdiploid (212 cases) and nonhyperdiploid (171 cases), respectively. More than 96 % of the respective genes were also found in the complete dataset. In addition, we investigated the impact on samples containing at least one of the high risk copy number alterations gain(1q21) or del(17p13) (189 cases). We found 4911 (96 % overlap) differentially expressed genes in this subset.

### Impact of “shipping delay” on molecular classification and risk prediction

Recently, GEP based molecular classifications of MM [5, 6] and risk predictors have been published [13–16]. In order to check whether “shipping delay” has a significant impact on the performance of these classifiers we compared the distribution of molecular or risk subgroups in shipped vs in-house samples. For these analyses we combined the HD4 and MM5 sets.

Whereas the molecular TC classification is based on the expression of D type cyclins and the type of the recurrent immunoglobulin heavy chain translocation [5], the UAMS classifier was developed using unsupervised hierarchical clustering of GEP data and recognizes seven molecular subgroups [6]. Both molecular classifications were generated using GEP data of in-house samples. We did not detect significant differences in the distribution of molecular subgroups between in-house and shipped samples in the combined set of HD4 and MM5 (Additional file 4: Table S3).

Applying the IFM-15 risk predictor that had been developed using shipped samples we found significantly more high risk patients in in-house samples compared to shipped samples (Table 2). In order to rule out random variation in the data we additionally investigated the distribution of IFM-15 scores in the HD4 and the MM5 set separately. Both the HD4 (21.6 % vs. 31.4 %) as well as the MM5 set (17.8 % vs. 38.6 %) showed lower numbers of IFM-15 high risk cases in shipped samples. By contrast, the GEP70 (developed using in-house samples) and the EMC-92 (generated using a mixture of in-house and shipped samples) signatures showed no significant distribution inequalities of high risk cases in in-house compared to shipped samples (Table 2). We found 7/15 IFM15 genes, 32/70 GEP70 genes and 41/92 EMC-92 genes to show significant differential expression between shipped and in-house samples (Additional file 5: Table S5).

**Table 2:** Influence of “shipping delay” on risk prediction

Data set	n	Signature	Risk in-house [%]	Risk in-house [%]	*P*
			High / low	High / low	
HD4 & MM5	573	IFM-15	36.1 / 63.9	19.0 / 81.0	<0.001
combined		GEP70	11.7 / 88.3	11.4 / 88.6	0.9
		EMC-92	9.1 / 90.9	12.2 / 87.8	0.3

**Fig. 2:**
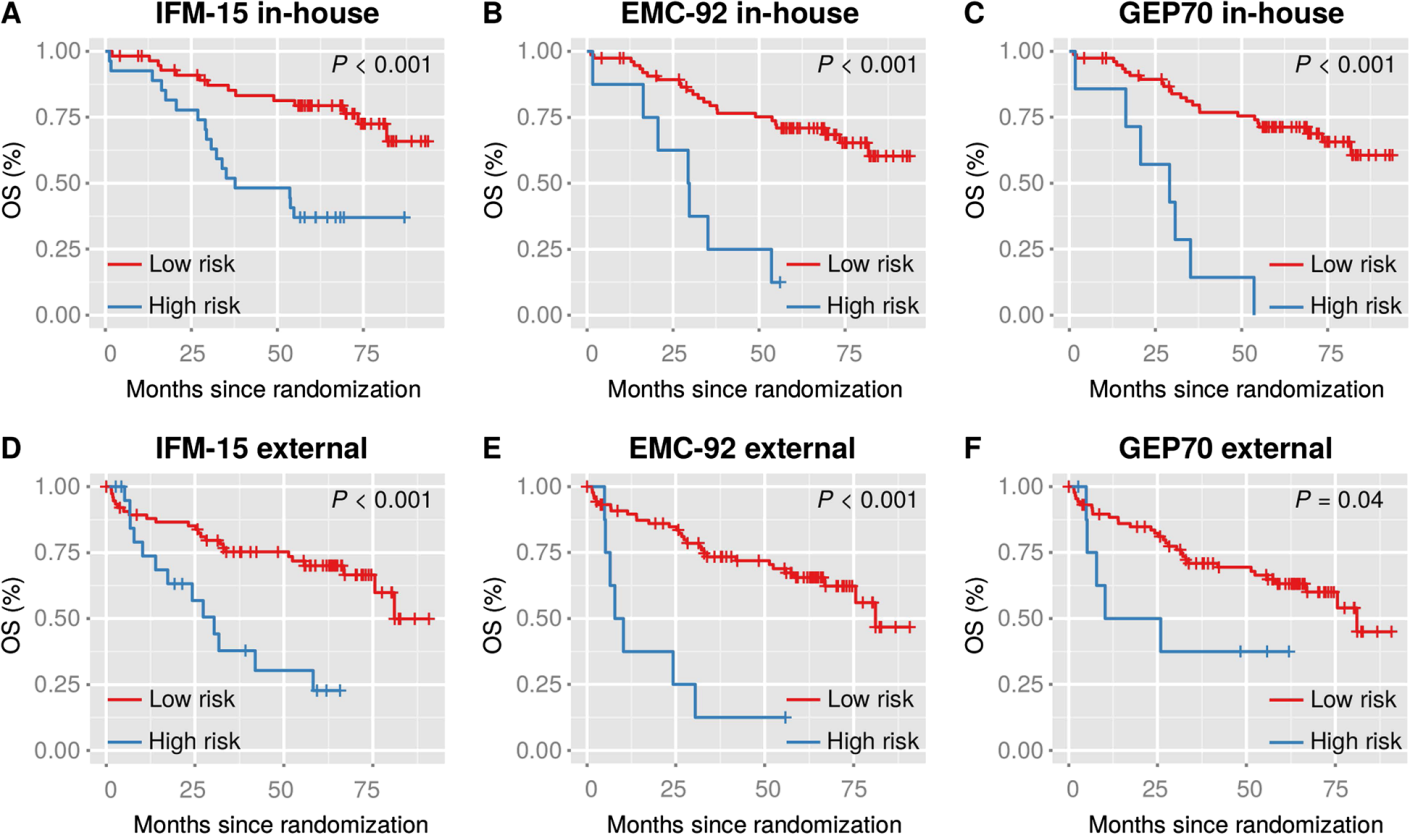
OS among MM patients according to risk and sample status. Kaplan-Meier analysis of OS is shown in relation to risk as determined by the IFM-15 (**a**, **d**), the EMC-92 (**b**, **e**) or the GEP70 (**c**, **f**) using in-house or external (shipped) samples of patients treated within the GMMG HD4 trial

To further investigate the impact of “shipping delay” on risk prediction we investigated OS of high and low risk HD4 patients as predicted by these signatures. As shown in Fig. 2, using GEP data from shipped samples all predictors still enabled the identification of patients with a significantly worse outcome. The impact of sample processing delay on the IFM-15 signature is further illustrated in Additional file 6: Figure S1. Although the overall survival was similar for patients treated in Heidelberg and external centers, low and high risk patients treated in external centers showed worse outcomes in comparison to the corresponding IFM-15 risk subgroups of in-house patients.

Next we analyzed whether inaccuracies introduced into gene expression profiling may weaken the value of the risk signatures to the extent that they are not independent of cytogenetic or clinical risk factors. We used Cox regression on OS of external HD4 patients including the prognostically unfavorable alterations gain(1q21) and del(17p13) and the international staging system (ISS) and one of the gene signatures respectively. The GEP70 did not show a significantly different prognostic effect (*P*=0.4,*HR*=1.64), whereas the IFM-15 (*P*=0.03,*HR*=2.49) and the EMC-92 (*P*=0.03,*HR*=3.67) were independently negatively associated with outcome (Additional file 7: Table S6).

### Prediction of shipped samples

We tested whether shipped samples can be identified based on their expression profile by applying PAM to the set of MM5 patients and validating the predictor with the HD4 set. The predictor consisted of 17 genes (Additional file 8: Table S4), all being part of the 3994 validated differential expressed genes. In the training set the overall error rate of the predictor, as tested by a 10-fold cross validation, was 0.06 (Table 3). Application of the predictor on the HD4 set resulted in an overall error rate of 0.15 (Table 3) confirming the accuracy of the predictor. The code to run the predictor is available on GitHub (https://github.com/meissnert/intext).

**Table 3:** 10-fold cross validation error rate and validation of 17-gene PAM predictor

	Training group (MM5)			Validation group (HD4)		
Status	Shipped	In-house	Class error	Shipped	In-house	Class error
			rate			rate
Shipped	237	9	0.04	73	24	0.25
In-house	15	130	0.10	4	81	0.05
	Overall error rate =0.06			Overall error rate =0.15		

**Fig. 3:**
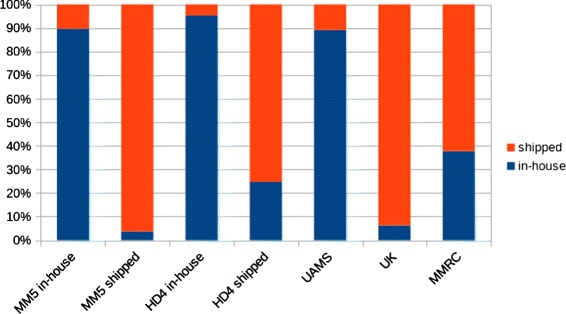
Prediction of sample status in different cohorts. Barplot depicting the percentage of samples predicted to be in-house (*blue*) or shipped (*red*) by the 17-gene PAM classifier. The MM5 (*bars 1 & 2*) and HD4 cohorts (*bars 3 & 4*) have been split into in-house and shipped samples according to the actual status

To further validate the predictor we applied it to three publicly available GEP datasets of newly diagnosed MM patients from the UK and the US (MMRC & UAMS). All samples in the UK dataset were sent to a central laboratory in the ICR (Sutton/UK) and served as a positive control for the identification of shipped samples. In contrast, we used the UAMS data as control set for in-house preparation of samples. The incubation time was not available for the MMRC set but according to a recent publication the MMRC standard operating procedure for sample shipment provides that samples are shipped at 4 °C [20]. In the UAMS set 498 of 559 samples (89 %) were predicted to be in-house (Fig. 3). In contrast 241 of 257 UK samples (94 %) were assigned to shipped samples. In the MMRC dataset 76 of 122 samples (62 %) showed the signature of shipped samples (Fig. 3).

## Discussion

GEP has been used by several groups for molecular analyses and risk stratification of MM. In multicenter MM trials BM aspirates are usually sent to a central processing laboratory for PC enrichment and sample storage as these techniques are not routinely available at all participating institutions, and building them up would imply a significant costs issue. Here we show that sample shipment has a profound impact on gene expression of MMPC.

More than one third of genes analyzed were significantly impacted by “shipping delay”. The effects were similar in cytogenetic subgroups of MM and samples showing the progression markers gain(1q21) or del(17p13) indicating that all subgroups of MM were equally impacted. Upregulated gene pathways included many signaling pathways, probably as an expression of cellular stress. On the other hand the downregulated pathways indicated slowing down of biosynthetic processes, as well as proliferation. Ahmann et al. investigated the impact of sample processing delay on gene expression in MM cells using paired bone marrow samples [20]. In contrast to our study the authors did not detect significantly differentially expressed genes in selected PC from shipped samples but this result was based on seven pairs only. Nevertheless, in that study eighty-two probe sets showed a 1.5-fold difference in expression level between immediately processed and shipped samples and an enrichment for pathways involved in responses to environmental stress. For 48 of these genes we found significant differential expression with fold changes up to 13.5 in our dataset as well (Additional file 2: Table S1). Another study that investigated BM samples reported that even short-term storage of these samples had a large impact on mRNA expression in unselected cells [21]. Only recently, a study using RNA sequencing data of hematopoietic cells showed that the impact of shipping was not confined to expression level differences but also changed RNA splicing and inhibited RNA surveillance [22]. These results and our findings indicate that GEP-based molecular analyses of MM need to take into account confounding by sample shipping.

Even more important, shipping may impact the performance of risk predictors. According to our data high risk cases as defined by the GEP70 and the ECM-92 signatures were evenly distributed in in-house and shipped samples. This suggests that the two predictors were resistant to the shipping effect, but more than 40 % of genes in them were significantly differentially expressed between in-house and external samples. Furthermore, in a Cox regression model on OS of external HD4 patients including the GEP70, the progression markers gain(1q21) and del(17p13) and the ISS, the GEP70 did not show an independent statistically significant prognostic effect. This result may be due to lacking statistical power, but may also indicate that inaccuracies introduced into GEP data weaken the value of this risk signature to the extent that it is not independent of cytogenetic or clinical risk factors anymore. In contrast, the EMC-92 signature developed on a mixture of in-house and external samples showed an independent prognostic impact on external samples. On the one hand, one could conclude that risk signatures developed on a mixture of samples perform better, but on the other hand this hypothesis cannot be proven using the HD4 dataset since the signature had been developed on it. Paired sample data were not available, preventing the investigation of the impact of sample processing delay on individual samples. In summary we cannot exclude at least minor effects on these predictors.

The IFM-15 risk signature showed a significantly different distribution in the two sets. As presented in Additional file 6: Figure S1 low and high risk patients treated in external centers showed worse outcomes in comparison to the corresponding IFM-15 risk subgroups of in-house patients. The IFM-15 risk signature is mainly composed of genes involved in proliferation. Our data show that GEP data of shipped samples were unsuitable for a reliable measurement of proliferation. The results suggest that proliferation was underestimated in external samples leading to a misclassification of patients whose increased risk was mainly due to higher proliferation rates of their tumor cells.

Despite the impact of the sample processing delay on gene expression, all three signatures still enabled the detection of patients with a significant worse outcome. This suggests that at least a subset of genes selected for high risk signatures may be an integral part of the high risk clone and expressed independently of environmental influences. Environmental independent gene expression could also explain why sample shipping showed no significant impact on molecular classification. Nevertheless, we recommend using in-house samples for discovery analyses as the “shipping effect” may obscure the true biology of MM cells.

Many centers involved in multi-center MM trials either do not have experience in plasma cell purification nor see the necessity to build this up, basically to avoid costs, and due to the fact that in most trials no clinical consequences are drawn from GEP based risk classifications. As a result, sample shipping cannot be avoided in these trials. Are there ways to avoid impact of shipping? There are commercially available RNA stabilizing reagents and tubes, but they cannot be combined with flow sorting protocols [23–25]. In blood cells the effect of sample processing delay on the transcriptome can be alleviated by incubating the cells on ice [22]. Applying our PAM predictor to the MMRC set, more than 60 % of samples were assigned to shipped samples, indicating that incubating BM samples at low temperatures before MM cell enrichment may not effectively reduce the effect of sample shipping on gene expression of MM cells. On the other hand, enrichment of MM cells at higher temperatures may have covered the positive effect of cooling samples during transport. Future studies will have to show whether cooling at transport and enrichment can reduce the negative impact of sample processing delay and if controlled shipping of cooled samples is feasible in multi-center MM trials. Based on available data, currently no clear circumvention of the shipping impact can be recommended.

How should one handle GEP based risk stratification within multi-center trials? The discussed results imply the following possibilities: to set up the MMPC purification at the participating centers, refer each patient before inclusion to a center with an experienced sample processing laboratory or accept inaccuracies in estimation of risk in shipped samples.

How should one interpret the existing MM GEP and RNAseq datasets and handle biorepositories? In publicly available datasets the origin of samples used for molecular analyses is usually not documented. We propose to apply our PAM predictor based on GEP data (limited to data derived from Affymetrix U133 Version 2.0 plus arrays) or the recently published panel of alternatively spliced exons for detection of impacted samples. These tools may suggest the samples to be excluded or the analysis being adjusted for this confounder. Alternatively,confounding effects may be identified and removed from expression data using the recently published “probabilistic estimation of expression residuals” [26].

## Conclusions

Our study shows that “shipping delay” widely influences gene expression of MMPC with different impact on molecular classification and risk stratification. It should be avoided if possible or at least be taken into account.

## Availability of supporting data

Predictor: https://github.com/meissnert/intext HD4 U133 2.0 dataset (GSE19784): http://www.ncbi.nlm.nih.gov/geo/query/acc.cgi?acc=GSE19784 MM5 U133 2.0 dataset (E-MTAB-2299): https://www.ebi.ac.uk/arrayexpress/experiments/E-MTAB-2299/ UK U133 2.0 dataset (GSE21349): http://www.ncbi.nlm.nih.gov/geo/query/acc.cgi?acc=GSE21349 UAMS U133 2.0 dataset (GSE24080): http://www.ncbi.nlm.nih.gov/geo/query/acc.cgi?acc=GSE24080 MMRC U133 2.0 dataset: http://www.broadinstitute.org/mmgp/home

## Additional files

Additional file 1
**Participating_centers.xls.** Names of the participating centers and the ethics committees that granted approval to this study. (XLS 35.5 KB)

Additional file 2
**Table S1 - Differentially Expressed Genes.** Table listing the 6802 genes differentially expressed between shipped and in-house samples. (XLSX 554 KB)

Additional file 3
**Table S2 - Altered Pathways.** KEGG Pathways significantly enriched in deregulated genes of shipped samples. (XLSX 11.5 KB)

Additional file 4
**Table S3 - Influence of ‘shipping delay’ on molecular classification of MM.** Distribution of molecular subgroups between in-house and shipped samples in the combined HD4 and MM5 set. (XLSX 10.9 KB)

Additional file 5
**Table S5 - Genes differentially expressed in risk predictors.** Subset of genes from IFM15, GEP70 & EMC-92 that show differential expression between shipped and in-house samples. (XLSX 16.3 KB)

Additional file 6
**Figure S1 - Impact of sample processing delay on IFM-15 signature.** Kaplan Meier plot depicting the impact of sample processing delay on the IFM-15 signature. (TIFF 805 KB)

Additional file 7
**Table S6 - Multivariate Cox regression on overall survival of HD4 patients.** Results of cox regression analysis on OS of external HD4 patients. (XLS 22.0 KB)

Additional file 8
**Table S4 - Genes of PAM predictor.** Table listing the 17 genes that make up the PAM predictor. (XLSX 9.86 KB)

## Abbreviations

BM: bone marrow
CDF: chip definition file
GEP: gene expression profiling
GMMG: German-speaking myeloma multicenter group
GPI: gene expression based proliferation index
IFM: the French intergroupe francophone du myelome
MM: multiple myeloma
MMPC: multiple myeloma plasma cells
MMRC: multiple myeloma research consortium
UAMS: The University of Arkansas for medical sciences
OS: overall survial
PAM: prediction analysis for microarrays
